# Accelerated Profile HMM Searches

**DOI:** 10.1371/journal.pcbi.1002195

**Published:** 2011-10-20

**Authors:** Sean R. Eddy

**Affiliations:** HHMI Janelia Farm Research Campus, Ashburn, Virginia, United States of America; University of Virginia, United States of America

## Abstract

Profile hidden Markov models (profile HMMs) and probabilistic inference methods have made important contributions to the theory of sequence database homology search. However, practical use of profile HMM methods has been hindered by the computational expense of existing software implementations. Here I describe an acceleration heuristic for profile HMMs, the “multiple segment Viterbi” (MSV) algorithm. The MSV algorithm computes an optimal sum of multiple ungapped local alignment segments using a striped vector-parallel approach previously described for fast Smith/Waterman alignment. MSV scores follow the same statistical distribution as gapped optimal local alignment scores, allowing rapid evaluation of significance of an MSV score and thus facilitating its use as a heuristic filter. I also describe a 20-fold acceleration of the standard profile HMM Forward/Backward algorithms using a method I call “sparse rescaling”. These methods are assembled in a pipeline in which high-scoring MSV hits are passed on for reanalysis with the full HMM Forward/Backward algorithm. This accelerated pipeline is implemented in the freely available HMMER3 software package. Performance benchmarks show that the use of the heuristic MSV filter sacrifices negligible sensitivity compared to unaccelerated profile HMM searches. HMMER3 is substantially more sensitive and 100- to 1000-fold faster than HMMER2. HMMER3 is now about as fast as BLAST for protein searches.

## Author Summary

Searching sequence databases is one of the most important applications in computational molecular biology. The main workhorse in the field is the BLAST suite of programs. Since the introduction of BLAST in the 1990's, important theoretical advances in homology search methodology have been made using probabilistic inference methods and hidden Markov models (HMMs). However, previous software implementations of these newer probabilistic methods were slower than BLAST by about 100-fold. This hindered their utility, because computation speed is so critical with the rapidly increasing size of modern sequence databases. Here I describe the acceleration methods I implemented in a new, freely available profile HMM software package, HMMER3. HMMER3 makes profile HMM searches about as fast as BLAST, while retaining the power of using probabilistic inference technology.

## Introduction

Sequence database homology searching is one of the most important applications in computational molecular biology. Genome sequences are being acquired rapidly for an ever-widening array of species. To make maximal use of sequence data, we want to maximize the power of computational sequence comparison tools to detect remote homologies between these sequences, to learn clues to their functions and evolutionary histories. The most widely used tool for sequence comparison and database search is BLAST [1]–[3].

Since BLAST's introduction, some important advances have been made in the theory of sequence comparison, particularly by using probabilistic inference methods based on profile hidden Markov models (profile HMMs) [4]. Probabilistic modeling approaches provide a consistent framework for parameterizing complex position-specific models of sequence conservation and evolution [5]. Numerous improvements have been made in BLAST in light of these advances [6]–[9]. Fundamentally, though, the BLAST implementation computes optimal local alignment scores using *ad hoc* gap penalties. This implementation core may not be readily adaptable to a probabilistic insertion/deletion model and the more powerful “Forward/Backward” HMM algorithm that computes not just one best-scoring alignment, but a sum of probabilities over the entire local alignment ensemble. The Forward algorithm allows a more powerful and formal log-likelihood score statistic to be assigned to each target sequence, and Forward/Backward allows confidence values to be assigned to each aligned residue.

Nonetheless, regardless of any of the attractive advantages of HMMs, no implementation of fully probabilistic sequence comparison methods has yet approached the utility of BLAST. The most widely used implementations of profile HMM technology, including HMMER from my laboratory, have been slow and computationally expensive, on the order of 100- to 1000-fold slower than BLAST for a comparably sized search. In an era of enormous sequence databases, this speed disadvantage outweighs any advantage of HMM methods. Profile HMM methods have become important only in the niche of protein domain family analysis, where the speed differential is compensated by being able to use a single profile HMM to represent a family of hundreds of homologous individual sequences [10], [11].

HMMER has been a target of many acceleration and optimization efforts [12]–[15] but these efforts have had limited impact. The only accelerations that have reported large gains have implemented HMMER's native dynamic programming algorithms on specialized hardware, including FPGAs (field-programmable gate arrays) [16]–[19], VLSI ASICs (special-purpose chips), GP-GPUs (general purpose graphics processor units) [20], [21], and large multiprocessor clusters [22], [23]. Fewer efforts have been made to develop fast heuristic profile HMM algorithms for standard commodity processors [24]–[26] in ways comparable to how BLAST heuristically approximates and accelerates Smith/Waterman optimal dynamic programming alignment [27]. The challenge is that to preserve the significant yet narrow gain in sensitivity that profile HMM methods show over BLAST [28]–[30], any useful profile HMM acceleration heuristic must be more sensitive than BLAST's already excellent heuristics.

Another reason for the limited impact of previous acceleration efforts is that they have almost exclusively focused on accelerating the optimal local alignment scoring algorithm (known as the Viterbi algorithm in the HMM literature) as opposed to the more desirable Forward algorithm. In part, this is because optimal local alignment algorithms are more well known, and in part it is because previous versions of HMMER itself implemented Viterbi rather than Forward scoring. Forward implementations are about 3- to 9-fold slower than Viterbi implementations, and the expected statistical distribution of Forward scores for profile HMMs was not understood well enough to assign accurate E-values (expectation values). I recently described a satisfactory solution to the latter problem [31], which leaves the problem of acceleration.

Here I describe the heuristic acceleration pipeline implemented by HMMER3, a reimplemented version of the HMMER software. In comparison to the previous version of HMMER, HMMER3 is about 100-fold faster because of the use of a new heuristic algorithm called the MSV filter, while also being significantly more powerful because it moves from optimal local Viterbi alignment to full Forward/Backward evaluation of alignment ensembles, exploiting more of the mathematical advantages of probabilistic modeling. Thus HMMER3 is now about as fast as BLAST, while extending the performance advantages of profile HMM methods.

## Results

### Overview

**Figure 1:**
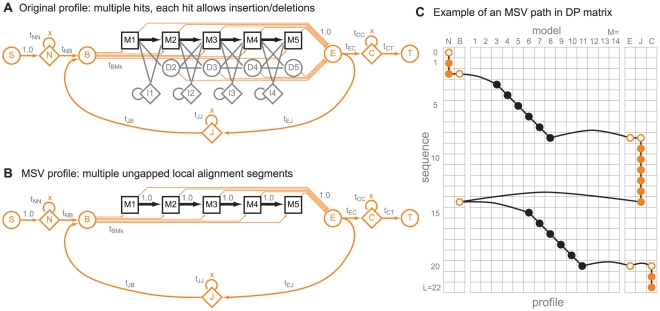
The MSV profile. A: Profile HMM architecture used by HMMER3 [4], [5], [31]. Regions homologously aligned to the query are represented by a linear core model consisting of 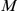 consensus positions (in this example, 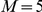), each consisting of a match, a delete, and an insert state (shown as boxes marked M, circles marked D, and diamonds marked I), connected by state transition probabilities (arrows). Match states carry position-specific emission probabilities for scoring residues at each consensus position. Insert states emit residues with emission probabilities identical to a background distribution. Additional flanking states (marked N, C, and J) emit zero or more residues from the background distribution, modeling nonhomologous regions preceding, following, or joining homologous regions aligned to the core model. Start (S), begin (B), end (E) and termination (T) states do not emit. B: The MSV profile is formed by implicitly treating all match-match transition probabilities as 1.0. This corresponds to the virtual removal of the delete and insert states. The rest of the profile parameterization stays the same. This model generates sequences containing one or more ungapped local alignment segments. Note that both models appear to be improperly normalized; for example, each match state in the MSV model has probability 1.0 local exit transition (orange arrows) in addition to the probability 1.0 match-match transition. This is because of a trick used to establish a uniform local fragment length distribution, in which these profiles are collapsed representations of a much larger (and properly normalized) “implicit probability model”, as explained in [31]. C: An example of what an alignment of a larger MSV profile (of length 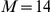) to a target sequence (of length 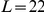) might look like, as a path through a dynamic programming (DP) matrix. Here, the model identifies two high-scoring ungapped alignment segments (black dots, indicating residues aligned to profile match states), and assigns all other residues to N, J, and C states in the model (orange dots; unfilled indicates a “mute” nonemitting state or state transition). Note that the ungapped diagonals are not enforced to be consistent with a single gapped alignment.

The main algorithm that accelerates HMMER3 is called MSV, for Multiple (local, ungapped) Segment Viterbi. It was inspired by a technique used in ParAlign [32]. As shown in Figure 1, the MSV model is an ungapped version of HMMER3's multihit local alignment model. MSV's probabilistic model of multihit ungapped local alignment is achieved simply by ignoring the match, delete, and insert state transitions of the original profile and implicitly treating match-match transitions as 1.0.

An MSV score is essentially analogous to BLAST's “sum score” of one or more ungapped HSPs (high scoring pairs). A difference is that MSV does not impose alignment consistency (two ungapped alignments are not required to be consistent with a single gapped alignment). In a filtering heuristic, this difference is not important. HMMER3 calculates the MSV score directly by dynamic programming, bypassing the word hit and hit extension heuristics of BLAST.

The fact that MSV essentially bypasses two of BLAST's main heuristics provides an intuitive argument why MSV scores are expected to be a more sensitive overall heuristic than BLAST's approach. However, I have not attempted to rigorously compare the performance of HMMER's MSV heuristic to other acceleration heuristics such as those in BLAST or FASTA.

The HMMER3 implementation takes advantage of several synergistic statistical and computational features of the MSV model. I summarize these features here before describing them in detail:

- MSV alignment scores can be calculated efficiently using so-called “striped” vector-parallel techniques originally developed for Smith/Waterman local sequence alignment [33], because the MSV model removes deletion and insertion states that interfere with vector parallelism.
- Because the MSV model gives predictable score distributions for nonhomologous sequences, with scores confined to a narrow range that is largely independent of query and target sequence characteristics, MSV values can be approximated with reduced precision (8 bits, in a score range of 0–255). This allows a 16-fold vector parallelism in current commodity processors with 128-bit vector registers.
- The MSV model remains a full probabilistic local alignment model, so MSV scores obey conjectures about the expected Gumbel distribution of probabilistic local alignment scores [31]. This allows the rapid calculation of P-values.
- Because we can calculate MSV P-values, we can use MSV scores as a tunable and selective sequence filter. If a target sequence has an MSV score with a P-value less than a chosen threshold, we pass the entire sequence to more accurate and computationally intensive scoring algorithms. By definition, the P-value threshold is the fraction of nonhomologous sequences expected to pass the filter.

The MSV filter is a heuristic acceleration, not guaranteed to find all high-scoring targets. Overall performance of the HMMER3 acceleration pipeline in terms of speed, specificity, and sensitivity depends on several issues and tradeoffs, including how fast the filters are, how accurately and quickly P-values can be estimated for filter scores, and whether a threshold on MSV P-values can be set to remove most nonhomologs while removing few if any true homologs that an unfiltered search would have detected. These are empirical questions, which I have addressed by benchmarking experiments.

The following sections, especially on vector parallelization and on assuring that scores can be kept in limited numeric ranges, are necessarily technical and terse. On a first reading, the reader may want to skip or skim ahead to the “HMMER3 acceleration pipeline” section to see how these technical aspects fit together into an overall scheme, and how that acceleration scheme performs.

### MSV model: notation and parameterization

The MSV score for target sequence 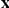 is a standard HMM Viterbi score, a log likelihood ratio score of a single optimal (maximally likely) alignment: the ratio of the probability of the optimal alignment 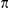
° for 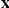 given the MSV model 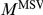 and the probability of the sequence given a null hypothesis model 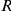:
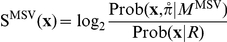
For a query of length 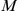 positions, the MSV profile has 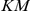 match emission parameters (where 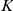 is the alphabet size, 4 nucleotides or 20 amino acids), plus 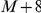 additional state transition parameters involving the flanking N, B, E, C, and J states that account for nonhomologous residues. Other state transitions in the original profile are ignored, which means implicitly treating match-match transitions as 1.0.

The null model 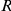 is assumed to be an HMM with a single state 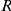 emitting residues 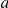 with background frequencies 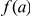 (i.e. a standard i.i.d. null model: independent, identically distributed residues), with a geometric length distribution specified by a transition parameter 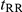.

The 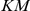 position-specific match scores 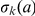 are precomputed as log-odds ratios for a residue 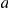 emitted from match state M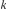 with emission probability 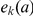, compared to the null model background frequencies 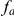:
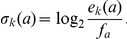
These match scores (as well as the emission probabilities and background frequencies) are the same as in the original profile.

The only state transition parameters in the MSV model are those that control target sequence length modeling, the uniform local alignment fragment length distribution, and the number of hits to the core homology model per target sequence [31]. These too are identical to the parameterization of the original profile [31]. Specifically, they are set as follows for a target sequence of length 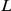 residues and a model of length 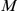 consensus positions:

Target sequence length modeling:
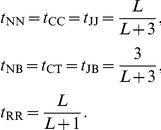



Uniform local alignment fragment length distribution:
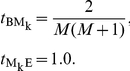



Multiple hits per target:
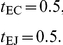



### MSV score algorithm (serial version)

The MSV alignment score can be calculated by a dynamic programming recursion in a two-dimensional matrix 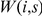 indexed by HMM state 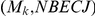 and target sequence residue 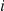:

Initialization:

 

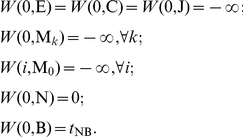



Recursion:

 

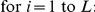



  

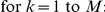



   

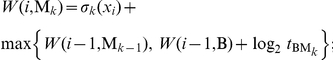



  

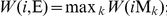



  

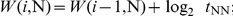



  

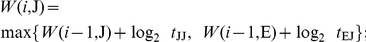



  

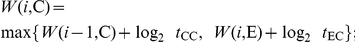



  

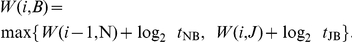



Termination: 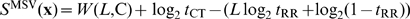


The 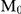 “state” in the initialization is solely needed for a boundary condition; there is no such state in the model.

Log-odds ratio scoring relative to the null model is built into the calculation, in the match scores 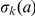 and in counting the total (constant) null model state transition contribution of 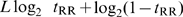 as terms in the DP termination step.

Like other linear sequence alignment recursions, the algorithm requires 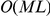 time. It is implemented in a single lattice row of 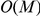 space for purposes of obtaining just the optimal score. In the HMMER3 source code, this algorithm is implemented in generic_msv.c::p7_GMSV().

### MSV score algorithm: SIMD vector parallelization

The MSV algorithm is highly amenable to vector parallelization using commodity SIMD (single instruction, multiple data) instructions, such as the Streaming SIMD Extensions (SSE) instructions on Intel-compatible systems and Altivec/VMX instructions on PowerPC systems. These vector instruction sets use 128-bit vectors to compute up to 16 simultaneous operations.

Several vector methods have been described for accelerating classical Smith/Waterman local sequence alignment [34], [35], and methods for accelerating Smith/Waterman dynamic programming (DP) recursions are readily adapted to profile HMMs. A remarkably efficient vector-parallel approach called *striped* Smith/Waterman was described by Farrar [33].

**Figure 2:**
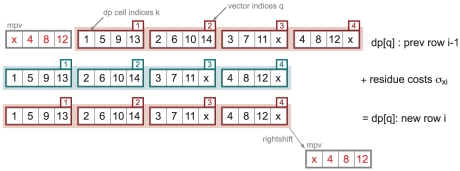
Illustration of striped indexing for SIMD vector calculations. The top row (magenta outline) shows one row of the dynamic programming lattice for a model of length 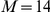. Assuming an example of vectors containing 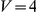 cells each, the 14 cells 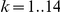 are contained in 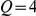 vectors numbered 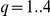. (Two unused cells, marked x, are set to a sentinel value.) In the dynamic programming recursion, when we calculate each new cell 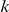 in a new row 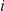, we access the value in cell 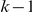 in the previous row 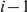. With striped indexing, vector 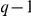 contains exactly the four 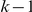 cells needed to calculate the four cells 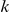 in a new vector 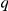 on a new row of the dynamic programming matrix (turquoise outline). For example, when we calculate cells 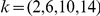 in vector 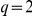, we access the previous row's vector 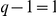 which contains the cells we need in the order we need them, 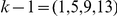 (dashed lines and box). If instead we indexed cells into vectors in the obvious way, in linear order (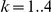 in vector 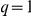 and so on), there is no such correspondence of 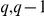 with four 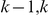
's, and each calculation of a new vector 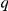 would require expensive meddling with the order of cells in the previous row's vectors. With striped indexing, only one shift operation is needed per row, outside the innermost loop: the last vector on each finished row is rightshifted (mpv, in grey with red cell 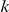 indices) and used to initialize the next row calculation.

Striping addresses a challenge in the data dependency pattern in Smith/Waterman-style dynamic programming recursions. The calculation of each cell 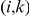 in the dynamic programming lattice requires having previously calculated cells 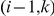, 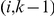, and 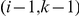. In a row-vectorized implementation, 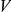 individual cells (typically 4, 8, or 16) are stored in each individual vector, such that each row 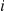 of the vectorized DP matrix stores cells 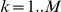 in Q vectors numbered 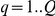, where 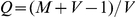. In Farrar's approach, cells 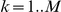 are assigned nonconsecutively to vectors 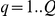 in a striped pattern (Figure 2). In striped vectors, when we calculate the set of several cells 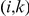 contained in one vector 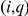 on a current row, all the previous diagonal cells 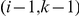 that we need are neatly available in the correct order in a vector 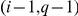 on the previous row, and the cells above are in vector 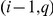. Striping minimizes expensive operations such as shifting or rearranging cell values inside vectors. The disadvantage is that calculations on delete paths (dependent on cells 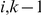 to the left) may need to be fully serialized. Farrar described effective techniques for minimizing this problem. In the MSV algorithm, because only ungapped diagonals are calculated, this drawback is avoided altogether. The essential idea of how striped indexing works is schematized in Figure 2.

To maximize parallelism, I implemented MSV as a 16-fold parallel calculation with score values stored as 8-bit unsigned integers restricted to range 0‥255. This takes advantage of the fact that local alignment scores under HMMER3's probabilistic model have a narrow and predictable dynamic range, enabling a numerical stability analysis that justifies using reduced precision. (The details of this analysis are given in the next section.) This rescaling is specified by three values (base, bias, and scale), where “base” is an initial offset from zero to make MSV scores nonnegative (default: 190), “scale” is the scaling factor (default 3, so MSV scores are in units of one-third bits), and “bias” is an offset on individual residue scores, used to make all individual residue scores unsigned byte costs relative to the maximum residue score. Using the scale and bias terms, position-specific residue scores 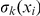 are converted to precomputed scaled costs by 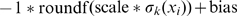 (saturated at a maximum cost of 255) and stored in striped order in vectors 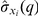 (Figure 2). Transition scores 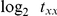 are converted to precomputed scaled costs 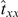 by 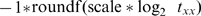 (saturated at a maximum cost of 255).

To define MSV's SIMD recursion, I will use five pseudocode vector instructions for operations on 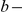
bit integers (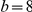 in our implementation), either scalars 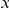 or vectors 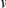 containing 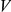 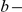
bit integer elements numbered 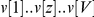. Each of these operations are either available or easily constructed in both SSE and Altivec/VMX:[Table pcbi-1002195-t001]

**Table pcbi-1002195-t001:** 

Operation	Pseudocode	Definition
saturated addition	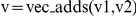	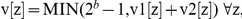
saturated subtraction	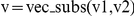	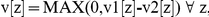
max	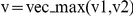	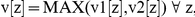
assignment	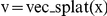	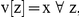
right shift	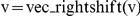	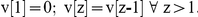
horizontal max	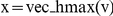	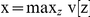

In this pseudocode, the vectorized MSV algorithm is the following:

Initialization:

 

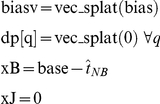



Recursion:

 

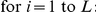



  

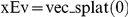



  

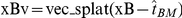



  

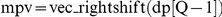



 

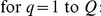



  

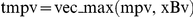



  

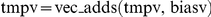



  

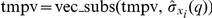



  

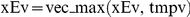



  

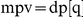



  

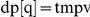



 

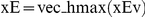



 

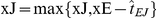



 

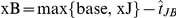



Termination: 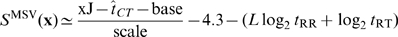


The constant term of −4.3 bits in the termination step arises from an approximation that deals with roundoff error in counting 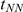 and 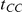 transition costs. This is explained in the following section. The termination condition is assuming that 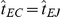, so that values for the C state are the same as for the J state (thus saving having to calculate C state values in the recursion).

This algorithm is implemented both for SSE and Altivec/VMX instructions in the HMMER3 source code in impl_{sse,vmx}/msvfilter.c::p7_MSVFilter().

### Analysis of consequences of reduced numerical precision

This section is particularly technical, and may be skipped in a first reading. In reducing the dynamic range of score calculations to small unsigned integers, we must make sure that underflow or overflow either do not occur, or have no erroneous consequences. We must also be sure that the magnitude of any accumulated roundoff error is tolerable. Because the HMMER3 acceleration pipeline (described below) uses vector-parallel, striped, reduced precision implementations of both the MSV algorithm (described above) and the standard Viterbi (optimal alignment) algorithm for the original profile model with insertions/deletions, the following analysis considers both MSV and Viterbi scores.

For underflow, we use the fact that there is a lower bound on optimal local alignment scores as a function of model length 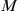 and target sequence length 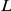. In the worst possible positive-scoring optimal local alignment, the core profile matches only one match state 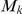 against one residue 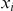 with a score 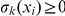, and the remaining 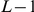 residues of the target sequence are accounted for by flanking N and C states. The worst case therefore has a log-odds likelihood score 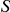 of
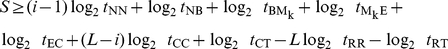
which is:
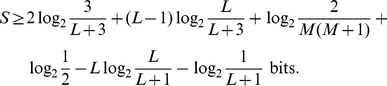
Known protein sequences can be over 30,000 residues long (human titin, for example, is 34,350aa). If we specify 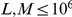 as design limits, we can assume a lower score bound of 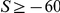 bits for optimal local alignments.

For managing overflow, we use the fact that we will only use a reduced-precision implementation as a filter on target sequences. Any sequence with a P-value
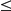
a chosen threshold will be passed on to a slower routine for recomputation at full precision. Using *saturated* arithmetic instructions, any target sequences that overflow will be scored as the highest possible score. Now we only need to be able to guarantee that the upper score bound has a P-value
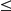
the lowest P-value threshold we ever plan to use. From Milosajevic [31], [36], we know a conservative bound 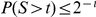 for a bit score threshold 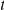. For a design limit allowing filter thresholds 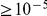, an upper score bound of 17 bits suffices. (0.02 is the default P-value threshold for MSV, and 0.001 is the default for Viterbi scores, as discussed below).

This range of −60 to 17 bits applies to *complete* optimal local alignments; in individual cells of the dynamic programming calculation, we need a little more dynamic range. A high scoring alignment of 17 bits, for example, will have a score of more than 17 bits in the last cell that aligns a match state to a homologous residue, because this state is always followed by negative scores from EC, CC, and CT transitions in the optimal alignment. Taking this into account (including some order of evaluation issues - the fact that the contributions of some transitions, including the null model's contributions, are included in a termination step after the dynamic programming recursion is complete) it can be shown that a range of −61…21 bits suffices to guarantee that no DP cell involved in an optimal local alignment of range −60…17 bits will underflow or overflow.

These same bounds apply to both the original local alignment model (Viterbi alignments with insertions and deletions) and the MSV model, because no step in ascertaining these bounds required any consideration of the transition probabilities in the core model (match, insertion, and delete states).

Thus we need a dynamic range of 82 bits (−61 … 21 bits), and the maximum range of an 8-bit integer is 256 values, so scaling log-odds scores to units of 1/3 bits suffices. (Coincidentally, this is comparable to the scaling and roundoff of standard scoring matrices used by BLAST or FASTA; BLOSUM45, for example, is in units of 1/3 bits.) A “base” offset term is then used to adjust the represented value range to the range of bit scores. For unsigned 8-bit integers, a base of +190 means that values 0‥255 represent the range of 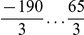 bits.

Rounding scores to the nearest 1/3 bit introduces a roundoff error of 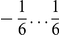 bit per scoring term. A sum of 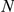 independent, identically distributed random deviates uniformly distributed on an interval 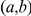 has mean zero and variance 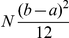. Because a local alignment score for a target sequence of length 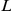 is modeled as a sum of 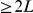 emission and transition scoring terms, even if each term's roundoff error were independent and uniformly distributed, accumulated roundoff error would be large (normally distributed with mean zero and variance 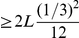; so for 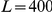 the accumulated error would have a standard deviation of 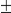
2.7 bits). Worse, roundoff errors are neither independent nor uniformly distributed. A particularly bad case is contributed by transition probabilities 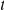 close to 1.0, such as most match-match transitions in the original gapped profile model, where 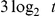 for all sufficiently large 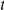 rounds to a zero cost. Another bad case is contributed by HMM states that have self-loops, such as insert-insert transitions, where a roundoff error is multiplied by the number of times the state is visited. These two bad cases make the self-loops producing chains of N, C, J, or insert states particularly problematic, because these self-transition probabilities are often close to 1.0; an entire chain of them often gets scored as zero, accumulating a large roundoff error.

The MSV model and its implementation use several features to reduce roundoff error to tolerable limits. First, by eliminating match, delete, and insert transitions and setting all match-match transition probabilities to 1.0 (thus zero cost), the MSV model itself has already eliminated many of the transitions that accumulate non-independent roundoff error, leaving in the core model only the 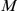 match state emission probabilities (which are all approximately independent and uncorrelated as far as roundoff error analysis is concerned). Second, the emission probabilities in N, C, and J states are assumed to be equal to the background (null model) frequencies, so the emission scores in N, C, and J are treated as zero by construction, thus they contribute no roundoff error terms. Third, we can take advantage of the fact that the total contribution of the NN, CC, and JJ transitions approximates a constant for sufficiently large 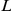, because a local alignment typically assigns nearly all residues of the target sequence to N, C, and J states and few to match states. Thus we expect a typical local alignment to involve on the order of 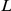 NN, CC, and JJ transitions, each scoring 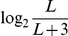, and 
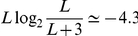
 bits for large 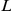. Therefore we can score NN, CC, and JJ transitions as zero cost during the recursion, then later add a constant 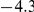 bits back onto the score to approximate their missing contribution. This approach may alter the optimal local alignment (during the recursion, paths using NN, CC, and JJ transitions look more favorable than they actually are) but in a score filter, we are not interested in the optimal alignment, only its score.

Thus the roundoff error in a reduced-precision MSV algorithm implementation consists of a bias arising from treating the NN, CC, JJ contributions totalling 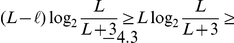 bits as a constant −4.3 bits, and a sum of more or less independent and uniformly distributed error terms including five or more log transition probabilities (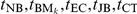) and 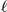 emission scores for match states involved in ungapped alignment diagonals of total length 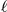 residues. For large 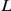, and assuming 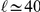 or so for typical MSV alignments at the edge of statistical significance, a back of the envelope calculation suggests an expected error of mean zero and standard deviation of about 
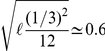
 bits, and a worst-case maximum error of about 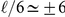 bits. Because higher-scoring alignments involve more match emission terms than low-scoring alignments, 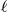 is correlated to the optimal score, so the roughly Gaussian error distribution for a given 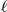 will be convolved with a Gumbel score distribution, resulting in a slightly non-Gaussian error distribution with some skew towards the higher error side.

To confirm that this expected roundoff error agrees with empirical observation, I performed simulations in which I examined the differences in MSV scores of a reduced precision implementation (unsigned bytes in 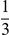 bit units) compared to a full-precision floating point implementation. I did this for many different profiles (9,318 models from Pfam release 22) aligned to 1,000 random sequences of varying lengths 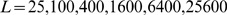, and to 207,132 real sequences in SwissProt 49.0 (in UniProt 7.0). This experiment showed roundoff errors for each model were distributed with a standard deviation of 0.4–0.6 bits for real sequences in Swissprot and for random sequences of each length. The mean error was approximately zero for random sequences of lengths 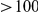; a 0.4 bit mean underestimate for UniProt sequences; a 0.5 bit mean underestimate for 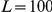 random sequences; and a 2.2 bit mean underestimate for 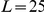 random sequences. On real UniProt sequences, I observed some extreme differences of up to 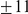 bits. These invariably corresponded to long and highly biased composition sequences, where presumably the alignment length 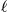 was large, increasing the potential for accumulated roundoff error. Because these were rare, and MSV is to be used only as a filter, these extremes seem safe to ignore. Overall the range of roundoff error appears tolerable, particularly for large 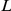.

### MSV scores obey conjectures allowing fast P-value determination

Previously [31] I conjectured that expected scores for certain probabilistic local alignment models, including the HMMER3 local alignment model, follow easily predictable distributions. Specifically, I conjectured that optimal alignment Viterbi bit scores show Gumbel distributions of fixed slope 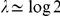, and the high scoring tail of Forward bit scores follow exponentials of the same slope 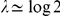. The MSV model is fully probabilistic and thus ought to obey these conjectures. Therefore MSV optimal alignment scores are predicted to follow a Gumbel distribution
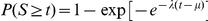
with slope 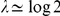 and a location 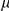 that is estimated by fitting to a small simulation of scores from 200 or so random sequences.

These statistical conjectures are best obeyed by models or scoring systems with high relative entropy per position (i.e. high mean expected score) [31]. Default HMMER3 models have low relative entropy per position (about 0.6 bits/position) because HMMER3 model parameterization uses a technique called entropy-weighting [29], [37], an *ad hoc* method to re-weight the effective number of observed sequences relative to the prior (pseudocounts) to achieve a desired relative entropy target. The standard pairwise residue alignment scoring system (BLOSUM62) has a similar relative entropy of about 0.6 bits per aligned position. At lower relative entropy per position, longer alignments are required to achieve high scores and a finite-length “edge effect” becomes considerable. HMMER3 ameliorates edge effect by calculating an *ad hoc* corrected 
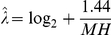
, where 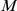 is the length of the profile in match states and 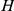 is the relative entropy per match emission state in bits [31]. Although the same *ad hoc* correction suffices for both Viterbi and Forward distributions, it was obtained by empirical fitting with little theoretical guidance, so there is little reason to trust that the same correction would apply to MSV scores. Therefore I empirically tested the ability to estimate accurate P-values for MSV scores for a wide range of HMMER/Pfam models.

**Figure 3:**
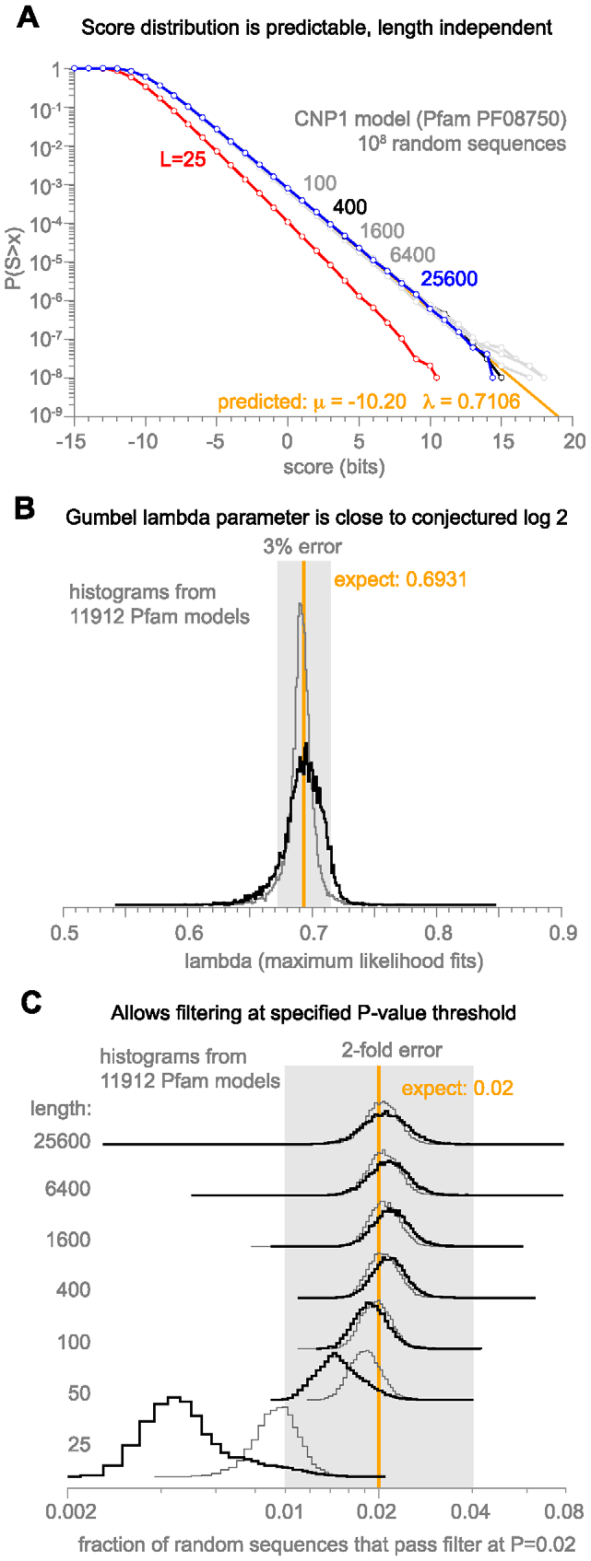
MSV scores follow a predictable distribution. A: example MSV score distributions for a typical Pfam model, CNP1, on 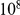 random i.i.d. sequences of varying lengths from 25 to 25,600, with the shortest, typical, and longest lengths highlighted as red, black, and blue lines, respectively. The predicted distribution, following the procedure of [31] including an edge correction on the slope 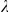, is shown in orange (though largely obscured by the data lines right on top of it). B: Histogram of maximum likelihood 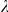 values obtained from score distributions of 11,912 Pfam models, showing that most are tolerably close to the conjectured 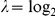, albeit with more dispersion for default entropy-weighted models (black line) than high relative entropy models without entropy-weighting (gray line). C: The observed fraction of nonhomologous sequences that pass the filter at a P-value of 0.02 should be 0.02. Histograms of the actual filter fraction for 11,912 different Pfam 24 models are shown, for a range of random sequence lengths from 25 to 25,600, for both default models (black lines) and high relative entropy models with no entropy weighting (gray lines).

Figure 3A shows an example of an MSV score distribution for one typical profile HMM (the CNP1 model from Pfam version 24, representing a lipoprotein family, chosen because it has the median length, median number of representative sequences, and median average pairwise identity over all Pfam 24 seed alignments), for 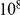 scores of random i.i.d. sequences of varying lengths. For all but the shortest sequences (L = 25), the observed score distributions closely match the conjectured distribution including the *ad hoc* edge correction term (orange line).

Figure 3B shows results of simulations in which 11,912 different profile HMMs from Pfam version 24 [11] were scored by the MSV algorithm against 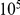 random sequences of length 400, the resulting distributions were fit to Gumbel distributions to determine maximum likelihood estimates of 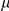 and 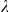, and a histogram of the 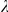 estimates is plotted. For high relative entropy models (grey line), this distribution is tightly clustered at the expected 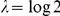. For default entropy-weighted models (black line), the distribution is broader with a higher mean, in accordance with what is observed for Viterbi scores and attributed to finite-length edge effect.

Figure 3C shows a direct evaluation of the accuracy of MSV P-values across many Pfam models and various random sequence lengths. For each of 11,912 Pfam 24 models, MSV scores are calculated for 500,000 random sequences generated at each of several lengths 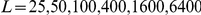 and 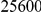, and the number of random sequences that pass the MSV filter at P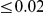 is counted. If P-values are accurate, we would expect to see an approximately normal distribution centered at 2% of random sequences passing the filter. Within a tolerance of about 2-fold error, this is true for almost all models and for target sequence lengths 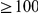 or so. A few models have less well-predicted distributions and produce modest outliers. The largest problems appear with short target sequences (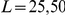) where P-values can be up to about five-fold overestimated (i.e., fewer sequences pass than predicted), as seen in the CNP1 example in panel A. Default entropy-weighted models (black) are more affected than models with high relative entropy (gray).

This analysis shows that in general, reasonably accurate P-values for MSV scores can be obtained. It also shows that on short sequences of 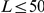 or so, the MSV filter may be too aggressive (removing more sequences than predicted), and that a few models are outliers with either too few or too many sequences getting through the filter. These are minor issues that would be good to deal with in the future.

### Vector parallelization of the Forward and Backward algorithms

The MSV implementation described above is about 500-fold faster than a standard serial implementation of the full Forward algorithm. This means that a search will still be rate-limited by the speed of the computationally intensive Forward/Backward calculations. Suppose we allow the top 2% (1/50) of sequences through the MSV filter to full HMM Forward log-likelihood scoring; then Forward must be no more than 50-fold slower than MSV, or Forward will be rate-limiting. It was therefore necessary to seek significant accelerations of at least an order of magnitude in the implementations of the Forward and Backward algorithms.

Numerical underflow is a problem for implementing the Forward and Backward algorithms. The probability of a partial alignment path generally underflows the smallest representable floating-point value. In a Viterbi implementation, underflow is avoided by working in the log probability domain, replacing multiplication and maximization of probabilities with addition and maximization of their logarithms [5], [38]. However, the Forward and Backward dynamic programming recursions require addition of partial paths in the probability domain.

In both sequence analysis and speech recognition HMM applications, this problem is customarily solved by working in the log probability domain and implementing a “log-sum” operation, such that addition 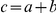 in the probability domain is replaced by 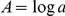, 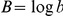, and 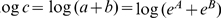 [5], [39]. An efficient log-sum operation rearranges the log-sum to 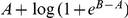 for 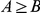, and finds an approximate 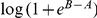 term to add to 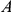 in a precalculated lookup table indexed by the difference 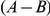 scaled and rounded to an appropriate precision. The lookup table has finite size, because the term is negligible for large 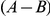, but nonetheless it is large (16,000 entries in the HMMER3 “generic” non-vectorized implementation; see logsum.c). I do not know how to implement a large lookup table efficiently in SIMD vector instructions. Only small lookup tables appear feasible using vector permutation instructions (up to perhaps 256 entries in Altivec/VMX, fewer in SSE).

Another approach is rescaling [5], [38]. In rescaling, the entries in each row 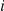 (for each target residue 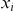) of the dynamic programming matrix are multiplied by some scale value 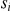. The scale values 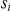 are chosen to keep the largest entries in each row within the allowable numeric range. If the smallest values in a row differ from the largest by greater than the numeric range, the smallest values still underflow, but for many HMMs one can show that no partial path prefix that underflows could have ever rebounded to have a non-negligible probability as a complete path. However, in general this is not the case for profile HMMs (nor for other HMMs with paths involving silent states), because of the possibility of long deletions. Even an optimal alignment can contain a long D-D-D path along a single row 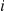. After many DD transition probabilities are multiplied together, the values in the states at the start versus the end of a long deletion path on the same row can differ by more than the allowable range. A standard normalized IEEE754 32-bit float type has a range of about 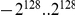, equivalent to 256 bits in the log-odds score domain. Given a typical deletion extension penalty of about −1 to −2 bits, a deletion of about 200 residues or so will typically underflow the rescaled delete states in the correct path. Deletions of this length are rare, but do occur.

I use the following steps to make an approach that I call “sparse rescaling” work for HMMER3's SIMD vector implementations of the Forward/Backward algorithms.

First, Forward/Backward values are calculated in an odds-ratio domain rather than the probability domain, so they are naturally pre-scaled to some extent. Each match emission probability 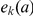 is replaced by its odds ratio 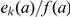, the same ratio used for log-odds scores. By the same arguments used to analyze underflow of the MSV implementation above, the odds ratio of the worst-case optimal local alignment path is a simple function of model and target lengths 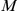 and 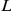, with a lower bound of about 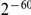 (about 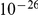) if we assume 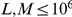 as design limits, well within the allowable range of an IEEE754 float representation.

Second, I exploit the fact that the HMMER3 profile HMM is a multihit local alignment model, not a glocal alignment model (glocal means global with respect to the model, local with respect to the target). When the profile HMM is a multihit local alignment model, a rescaling approach works. For any path with two aligned regions connected by a long deletion, there must exist an alternative path that counts the same two aligned regions as two local alignments, connected by a reinitiation path (M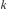 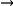
E
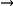
J
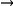
B). This reinitiation path, with only three transition probabilities on the same row, presents no underflow difficulties. The path with a long deletion may underflow, but then its complete path must be negligible relative to the alternative multihit local alignment path.

Determining the appropriate scale factor 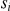 requires examining each value in the row, which typically requires extra computation. I exploit the fact that the value in the cell for the E state has already calculated a maximum over all 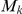 states. It is sufficient to use the E cell value itself as the scale value 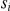, setting the E cell value to 1 and rescaling all other values in the row.

Still, rescaling a row also requires extra computation. Here I exploit the fact that rescaling every row is unnecessary. Instead, when the E cell odds-ratio value exceeds a certain threshold, this triggers a rescaling event for that row. Other rows have 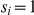.

I implemented Forward and Backward using sparse rescaling and striped SIMD vectors of four parallel 32-bit floats. Overall these implementations are about 16-fold faster than standard serial implementations using the log-sum lookup table operation. The overall 16-fold acceleration is likely a combination of about a 4-fold speedup from the SIMD vector parallelization with about 4-fold from replacing the log-sum operation with addition and multiplication. This makes the Forward/Backward algorithms only about 30-fold slower than the MSV filter.

### The HMMER3 acceleration pipeline

**Figure 4:**
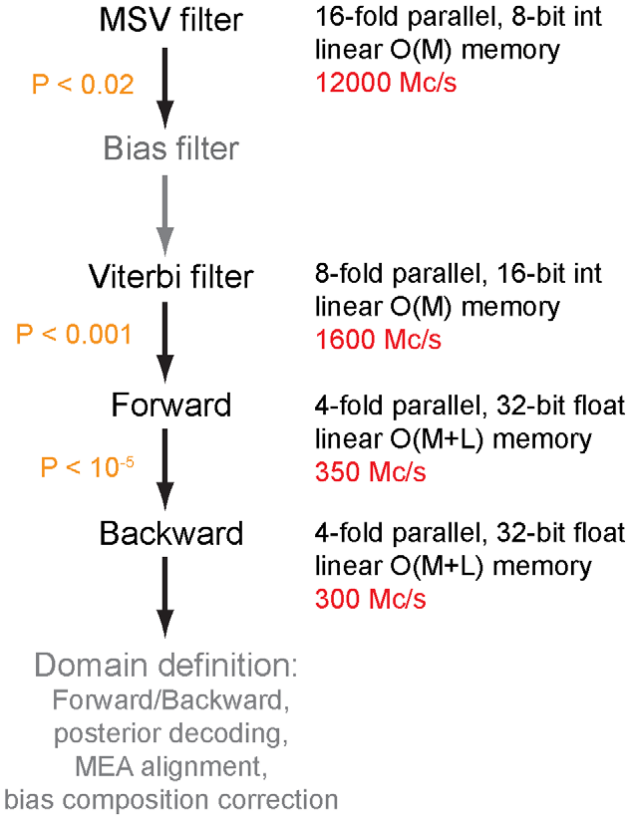
The HMMER3 acceleration pipeline. Representative calculation speeds are shown in red, in units of millions of dynamic programming cells per second (Mc/s). Default P-value thresholds for MSV, Viterbi, and Forward filtering steps are shown in orange. The bias filter and the domain definition steps are not described in detail in this manuscript, and are shown in gray.

The MSV and Forward/Backward methods described above are implemented in the so-called “acceleration pipeline” at the core of the HMMER3 software implementation (http://hmmer.janelia.org). The acceleration pipeline is summarized in Figure 4. One call to the p7_Pipeline() function is executed for each model/sequence comparison.

The pipeline either accepts or rejects the entire comparison at each step, based on the P-value of the log-odds score. For example, by default the MSV filter passes if a comparison gets a P-value of less than 0.02 (i.e., the top-scoring 2% of random nonhomologous sequences are expected to pass the filter). I have not yet explored the more sophisticated approach of using alignment information from earlier and faster steps in the pipeline to constrain (band) subsequent dynamic programming calculations.

All P-value calculations assume that the query profile and target sequence have residue compositions close to the overall average for proteins. In some cases, a query profile has a biased composition, and this bias matches a bias found in many target database sequences. Membrane proteins, for example, are skewed towards a more hydrophobic composition, and tend to match other nonhomologous membrane proteins with scores higher than expected under a simple average-composition null hypothesis. HMMER3 has methodology for recalculating scores and P-values to compensate for biased composition, but this methodology (the so-called “null2” correction, not described here for reasons of space) is placed late in the pipeline because it is computationally intensive. At the MSV filter step, the uncorrected MSV P-value may be underestimated in biased composition matches, which means more than the expected fraction of nonhomologous sequences may passes the MSV filter, which in some cases can be sufficient to slow the pipeline. HMMER3 inserts a “bias filter” step to reduce this problem. The bias filter step is shown in gray in Figure 4 because it is not described in detail in this paper. Briefly, the bias filter calculates a fast, heuristic biased composition correction to the MSV filter score using a two-state hidden Markov model with one emission distribution set to the average protein residue composition and the other emission distribution set to the average composition of the query profile, fully connected by four arbitrary hand-tuned transition probabilities. The pipeline rescores the sequence with this correction applied, and retests the modified P-value against the MSV filter threshold of 0.02. The bias filter has no effect on the final reported score of a sequence, which is calculated by the full Forward algorithm; the bias filter only has the effect of making the MSV filter remove additional matches that appear to be due to biased composition.

To further reduce the computational load that arrives at the full Forward step, an additional filter, the Viterbi filter, was implemented and inserted in the pipeline. The Viterbi filter is a striped SIMD vector implementation of optimal gapped alignment to the profile (Figure 1A). It is implemented 8-fold vector parallel in 16-bit integers, because the numerical analysis of roundoff error accumulation is less favorable for Viterbi than for MSV and more precision is required. Following the same arguments as described for MSV, Viterbi filter scores do not underflow (within design limits of 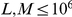) but may overflow the 16-bit representation, which is in units of 1/500 bits with an integer offset of 12,000, such that representable scores range from −89.5 to 41.5 bits. Any score that overflows the 41.5 bit upper limit is sure to pass any reasonable filter P-value anyway (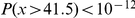; the default Viterbi filter threshold is 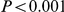).

The Forward and Backward algorithms for the pipeline are implemented in specialized efficient-memory forms called p7_ForwardParser() and p7_BackwardParser(). Each parser stores only a single row 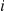 of the dynamic programming matrix for each sequence residue 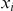, plus the complete columns for the “special” states E, N, J, B, and C. This yields 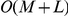 linear-memory implementations, with sufficient stored information to allow Forward/Backward posterior decoding of the probable positions of B and E states on the target sequence, defining the probabilities of local alignment endpoints. A target sequence that passes the Viterbi filter is scored with the full Forward parsing algorithm. If the Forward score passes a P-value threshold (default P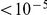), the Backward parser is calculated. Forward/Backward probabilities are used to estimate local alignment “regions” of substantial posterior probability mass in the target sequence. Each region is then subjected to a conceptually separate analysis pipeline, the “domain definition” pipeline, which identifies individual homologous regions and alignments, using a series of steps including full-matrix Forward/Backward, posterior decoding, maximum expected accuracy alignment, and a region-specific biased composition score correction. The domain definition procedure is not described in detail in this paper.

The acceleration pipeline is memory efficient. The MSV and Viterbi filters are only concerned with scores, not alignments, so they are implemented in linear-memory 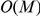 forms that store only a single dynamic programming row. The Forward and Backward algorithms are used in a 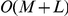 “parser” form just described. The domain definition pipeline, however, is not memory efficient. It currently calculates full 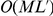 Forward/Backward and posterior decoding matrices for each identified subsequence (region) of length 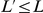 in the target sequence. Until these steps in the domain postprocessor are replaced with more memory-efficient algorithms, HMMER3 can occasionally exhaust available memory on some large model/sequence comparisons.

### Speed benchmarking

**Figure 5:**
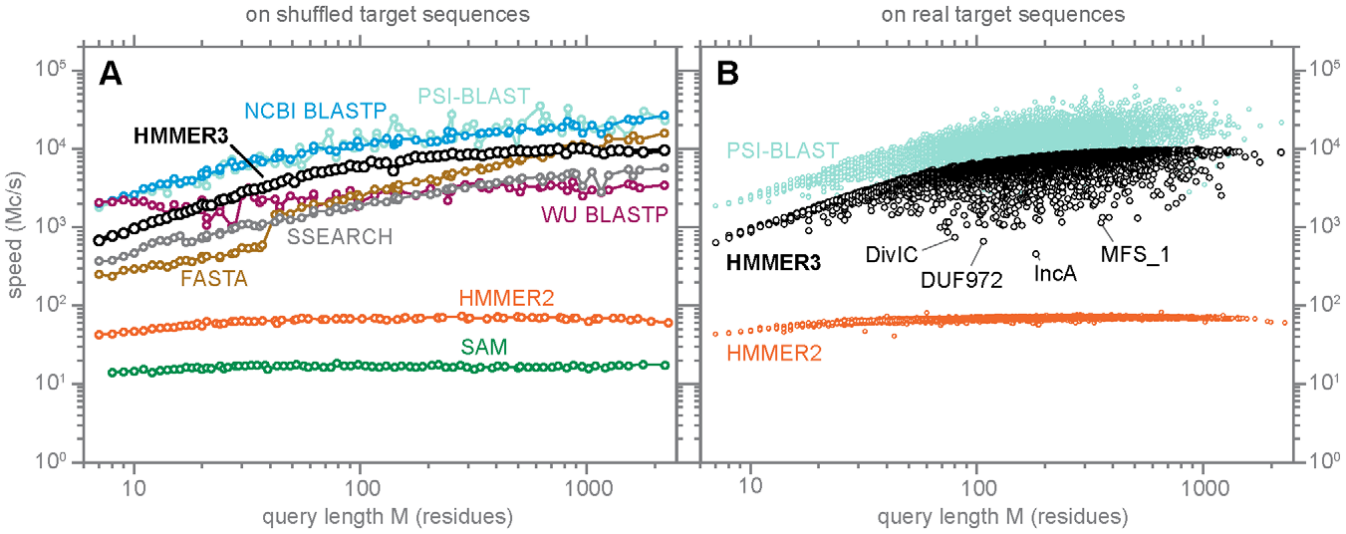
Speed benchmarks. Each point represents a speed measurement for one search with one query against 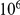 target sequences (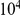 for the slow HMMER2 and SAM programs, 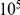 for FASTA and SSEARCH), on a single CPU core (see Methods for more details). Both axes are logarithmic, for speed in millions of dynamic programming cells per second (Mc/s) on the y-axis and query length in residues on the x-axis. Panel A shows “typical best performance” speed measurements for several different programs including HMMER3, for 76 queries of varying consensus lengths, chosen from Pfam 24, for searches of randomized (shuffled) target sequences. Panel B shows a wider range of more realistic speed measurements for all 11,912 profiles in Pfam 24, on searches of real target protein sequences from UniProt TrEMBL.

Figure 5 shows benchmark measurements of the speed of HMMER3, compared to the speed of BLAST [3], FASTA [40], SSEARCH (the FASTA implementation of Smith/Waterman), HMMER2, and the UCSC SAM profile HMM software [37]. Search speeds are shown in units of millions of dynamic programming cells calculated per second (Mc/s), measured on a single processor core (see Methods). The number of dynamic programming cells is the product of the query length 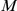 and the target database length 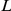 in residues. A straightforward implementation of dynamic programming sequence alignment scales in time as 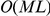, so reporting a speed in units of Mc/s is expected to be relatively independent of query and target length. In practice, the fastest search programs tend to show some additional dependence on query sequence length, with more efficient performance on longer queries. Figure 5 looks at a range of different queries of different lengths.

To measure the “typical” performance of each program, without complicating variation arising from producing the voluminous alignment output for some queries that hit large protein superfamilies, panel A (left) shows benchmarks done on random (shuffled) target sequences. The panel shows results for 76 query profiles (or representative single sequences), chosen to sample the full range of query lengths in the Pfam protein domain database from 7 to 2,217 residues. These results show that HMMER3 performance is comparable to other fast database search programs; somewhat slower (by about 2- to 3-fold) than NCBI BLAST, and somewhat faster (by about 3-fold) than WU-BLAST, for example. The speed of SSEARCH, the Smith/Waterman local alignment implementation in the FASTA package, is worth noting in this figure; SSEARCH has recently been accelerated by implementing Farrar's striped SIMD vector methods, allowing it to achieve speed comparable to the heuristic FASTA and WU-BLAST programs. HMMER3 is faster than HMMER2 by up to 140×, even though HMMER3 calculates full Forward scores whereas HMMER2 calculated faster Viterbi optimal alignment scores. Compared to SAM, which does calculate full Forward scores, HMMER3 is about 600× faster.

To measure a wider and more realistic range of real-world performance, panel B (right) shows benchmarks for 11,912 different queries (every Pfam 24 profile) on real sequences from UniProt TrEMBL. Programs that simply do a dynamic programming alignment to each target sequence, such as HMMER2 (orange points) or SSEARCH (not shown), show performance essentially independent of the properties of the query and target sequences. Programs that use heuristics and filters, however, are sensitive to how well a given search obeys the assumptions of the heuristic and/or filter thresholds. Both HMMER3 and PSI-BLAST speed vary not only by query length, but also vary substantially around their average for a given query length. PSI-BLAST speed in panel B varies both up (by up to about 3-fold) and down (by up to about 10-fold) from its “typical” performance in panel A, presumably reflecting variation in how many word hits and hit extensions need to be processed for a given search. HMMER3 speed tends to vary only downwards from its typical performance, by up to about 20-fold. In panel B, I highlight examples of four poorest-performing HMMER3 searches, on the DivIC, DUF972, IncA, and MFS_1 models. Even with the bias filter step included in the acceleration pipeline, the dominant cause of poorer HMMER3 search performance remains biased composition sequences (such as transmembrane proteins) in which more comparisons pass the fast filters of the acceleration pipeline than expected by P-value calculations that assume average target sequence compositions, causing more comparisons to reach the compute-intensive Forward/Backward calculations.

### Sensitivity/specificity benchmarking

**Figure 6:**
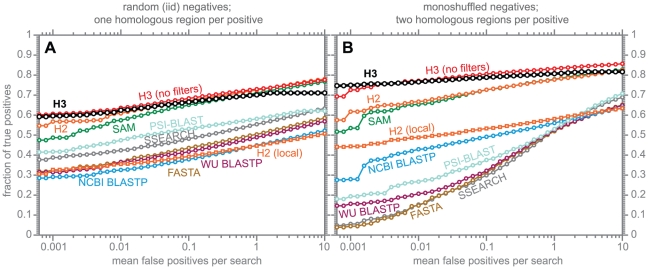
Benchmark of search sensitivity and specificity. For different programs, searches are performed either by constructing a single profile from the query alignment (HMMER3, HMMER2, SAM, PSI-BLAST), or by using “family pairwise search” [41] in which each individual sequence is used as a query and the best E-value per target sequence is recorded (BLASTP, SSEARCH, FASTA). In each benchmark, true positive subsequences have been selected to be no more than 25% identical to any sequence in the query alignment (see Methods). Panel A shows results where nonhomologous sequence has been synthesized by a simple random model, and each true positive sequence contains a single embedded homologous subsequence (a total of 2,141 query multiple alignments, 11,547 true positive sequences, and 200,000 decoys). Panel B shows results where nonhomologous sequence is synthesized by shuffling randomly chosen subsequences from UniProt, and each true positive contains two embedded homologous subsequences (a total of 2,141 query alignments, 24,040 true positive sequences, and 200,000 decoys). The Y-axis is the fraction of true positives detected with an E-value better than the number of false positives per query specified on the X-axis.

A filtering approach will generally compromise search sensitivity by some degree, because a filter will erroneously remove true homologs at some rate. We want this rate to be negligible. To measure how much search sensitivity is attenuated by the use of the MSV filter and the HMMER3 acceleration pipeline, I performed benchmarks to compare sensitivity/specificity of default HMMER3 hmmsearch (with the acceleration filter pipeline) to hmmsearch --max, an option that turns off all the filters and runs the full Forward scoring algorithm on every target sequence. I also benchmarked HMMER2 and several other homology search programs for comparison. These results are shown in Figure 6.

These benchmarks are automatically and semi-randomly generated by a program (create-profmark). The program starts from a source of trusted alignments of homologous protein domains (here, Pfam 24 seed alignments), a source of typical full-length protein sequences (here, UniProt SwissProt 2011_03), and a choice of method for synthesizing nonhomologous sequence segments (such as shuffling a randomly chosen segment of a UniProt sequence). A query alignment and a set of true test domains (trusted to be homologous to the query) is created by applying single-linkage-clustering by percent identity to a Pfam alignment, and using that clustering to select sequences such that no true test domain has more than 25% pairwise identity to any sequence in the query alignment, and no more than 50% pairwise identity to any other test domain. True test sequences are created by concatenating one or two test domains together with nonhomologous sequence segments, with a total sequence length sampled from the distribution of UniProt sequences. False (nonhomologous) test sequences are created by concatenating nonhomologous sequence segments, with both segment length and total sequence length sampled from the length distributions of the true test sequences. The procedure, its rationale, and some of its caveats are described in more detail in Methods.

To benchmark a profile method (HMMER, SAM, or PSI-BLAST), a profile is built from each query alignment and searched against the target database of test sequences and decoys. The results of all searches (for all different queries) are merged and sorted by E-value, and this ranked list is used to calculate a plot of fraction of true positives detected at increasing thresholds of false positives per query from 0.001 to 10.

To benchmark a single-sequence query method (BLAST, SSEARCH, FASTA) more fairly against profile methods, a family-pairwise-search (FPS) method is used [41] (as opposed to selecting just one query sequence from the alignment). Each individual sequence in the query alignment is searched against the target database; the best E-value found to any query sequence is treated as the E-value of the target sequence, and results for all queries are merged, sorted, and treated as above.

Figure 6 shows results for two different benchmarks. Panel A shows results where true test sequences have a single embedded domain, and nonhomologous sequences are synthesized as i.i.d. (independent identically distributed) random sequence from the average UniProt residue frequency distribution; there are 2,141 query alignments, 11,547 true test sequences, and 200,000 decoys. Because nonhomologous sequences are unrealistically simple (no biased composition, no repetitive sequence), this benchmark does not exercise the various corrections for biased composition that some programs have (such as HMMER and BLAST), and is more of a baseline test of the best-case sensitivity of a search program. Panel B shows results of my default profmark benchmark, where there are two embedded domains per true test sequence (in order to test the ability of a program to correctly detect and align multiple domains per sequence, although such results are not shown here), and where nonhomologous sequence segments are created by shuffling randomly chosen segments of UniProt sequences. Because nonhomologous segments in this benchmark can show more realistic monoresidue composition biases (though not higher-order bias such as tandem repeats), this version of the benchmark is more realistic and exercises more *ad hoc* parts of programs that try to correct for biased composition. Panel A shows results that are largely independent of biased composition issues, but less realistic. Panel B shows results that are more dependent on the ability of a program to handle biased composition, and probably more realistic.

The main result in both panels is that sensitivity and specificity are essentially identical for HMMER3 either with the acceleration pipeline (dark black lines) and without it (--max; red lines). There is a slight loss in sensitivity caused by the acceleration pipeline, but this loss is more than compensated by the gain in sensitivity of HMMER3 over HMMER2 (either in its default “glocal” alignment search mode or its local alignment search mode; orange circles and squares respectively). At high specificity (low false positive rates) on more realistic biased decoys, default HMMER3 can appear to be better than unfiltered HMMER3 (hmmsearch --max) because the bias filter removes some problematic biased false positive decoys that the supposedly more powerful biased composition corrections in HMMER3 Forward scores fail to correct.

Benchmarks for other programs (such as BLAST, SAM, FASTA, and SSEARCH) are shown only for the sake of rough comparison. The intent is not to thoroughly benchmark HMMER against these programs, but to provide an additional sense of scale, putting the difference between HMMER3 with and without its acceleration pipeline in context – that is, showing that the difference between HMMER3 with and without its acceleration pipeline is minor, compared to differences among programs. These programs were only run in their default configuration. I did not explore available options that might improve their performance on this benchmark. Although I believe the results to be fair and representative, I have to interpret these results with caution. I benchmark HMMER routinely on these benchmarks during development. It is impossible to avoid some degree of training on the benchmark, even though the benchmarks are somewhat randomized. Nonetheless, some informative trends in these results agree with previous independent published benchmarking from other groups, so the comparisons are probably useful as a rough guide. For example, Madera and Gough published a benchmark [28] in which they concluded that SAM significantly outperformed HMMER2, which at the time disagreed with my experience. Johnson [29] traced this to the fact that Madera and Gough had switched HMMER2 from its default glocal alignment mode to its nondefault local search mode, which we had not spent much time testing or tuning, and we had not previously realized how sensitive local alignment is to the model's information content per position. This led us to realize how important SAM's entropy-weighting technique is for local alignment, whereas it is much less important in glocal alignment [29]. This story is reflected in the benchmarks in Figure 6, where HMMER2 *local* alignment performs poorly relative to SAM, HMMER2 *glocal* alignment is comparable to SAM, and HMMER3 local alignment (with the entropy-weighting technique) is perhaps a bit better than SAM. Most of the difference between HMMER3 and SAM is in the high-specificity regime of the more realistic benchmark that includes biased-composition segments (Panel B), and thus is likely to result from differences in the *ad hoc* bias composition corrections that differ between SAM and HMMER, rather than any fundamental difference in their profile HMM parameterizations or their Forward scoring algorithms, which I believe are quite similar.

Another story reflected in these benchmarks is about the widespread belief that full Smith/Waterman alignment is superior to BLAST's fast heuristic approximation of Smith/Waterman. This is true in the easier benchmark (Panel A) but not when decoy sequences include biased composition segments (Panel B). Indeed, in Panel A all three fast sequence search programs (WU-BLAST, NCBI-BLAST, and FASTA) perform comparably (and worse than SSEARCH), whereas in Panel B, NCBI BLAST outperforms WU-BLAST, FASTA, and SSEARCH. This again seems likely to be showing the importance of biased composition score corrections. Biased composition correction has received close attention in NCBI BLAST software development [7], [8], [42], but is not part of the textbook description of “optimal” Smith/Waterman local alignment.

My main conclusion (that the acceleration pipeline has a negligible impact on the sensitivity/specificity of HMMER3 compared to unaccelerated Forward scoring) is supported by a more direct experiment. I searched all 11,912 Pfam 24 profile HMMs against the 516,081 sequences in UniProt SwissProt 2011_03 using five different option settings to hmmsearch, starting with --max and then successively turning on one filter step at a time in the acceleration pipeline (MSV, bias, Viterbi, and Forward), up to the default configuration with all four filter steps on. With --max, a total of 799,893 hits were found with an E-value of 0.0001 or less. Turning on the MSV filter loses 718 hits (0.09%). Overall, the default pipeline with all filter steps loses 2,701 hits (0.3%). Differences in significant hits are not necessarily all due to true homologs. It is possible for the unfiltered search to find a false positive that one or more of the filters would remove. However, the majority of these differences appear to be true homologs that are removed by the filters. Other than the 718 hits removed by the MSV filter, the great majority of the other losses are due to the bias filter inappropriately removing sequences that have strong biased compositions, but also contain a true homology region. The most obvious problems with HMMER3 sensitivity/specificity seem to lie in its bias filter and bias composition score corrections, rather than in the use of the MSV filter as its primary acceleration.

## Discussion

In describing the MSV heuristic and other acceleration methods implemented in HMMER3, I have not addressed the question of whether the MSV heuristic is better or worse than other heuristics, such as those in BLAST or FASTA. In sensitivity/specificity benchmarks (Figure 6), BLAST and FASTA perform about the same as unaccelerated Smith/Waterman, and HMMER3 performs about the same with and without its acceleration pipeline. This show that the overall sensitivity and specificity of these programs are not limited by their respective heuristics, but rather by their fundamental (unaccelerated) sequence comparison methods. Thus a better heuristic would be unlikely to improve overall sensitivity/specificity. A better heuristic would be a faster one with the same sensitivity/specificity. In a question about speed, we cannot satisfactorily show that one algorithm is necessarily faster than another. We can only rigorously compare particular implementations. Trying to mix and match different heuristic approaches and make definitive comparisons of their fully optimized speeds requires a time-consuming engineering and optimization effort dedicated to each implementation. So it remains an open question whether, for example, BLAST-style heuristics could be tuned to have enough sensitivity/specificity to match HMMER3 performance while still being faster. I developed MSV because it was easy for me, not because I tried different heuristics and found MSV to be better. I was working on striped SIMD vectorization of the Viterbi algorithm, and an MSV implementation is easily derived from striped SIMD Viterbi just by deleting the code that handles deletions and insertions. The heuristic approaches in BLAST and FASTA have the advantage of focusing subsequent slower computations on particular diagonals, whereas in HMMER's current approach, we wastefully recalculate full sequence alignments at each step of the acceleration pipeline. I expect that it will be fruitful to develop heuristics focused around high-likelihood diagonals, as BLAST and FASTA do, while using HMMER's SIMD vectorization methods.

Although this paper is about the acceleration methods used in HMMER3, HMMER3 also appears to be more sensitive than HMMER2. The main reason for this is the adoption of “entropy-weighting”, a method introduced by the UC Santa Cruz group in the SAM profile HMM package [29], [37], where the information content per position is reduced to a specified target number of bits. A second reason is the switch from Viterbi optimal alignment scores to Forward scores summed over the alignment ensemble [29].

On the other hand, I believe that the switch from default glocal alignment in HMMER2 to local alignment in HMMER3 has probably compromised some search sensitivity (“glocal” means global in the query, local in the target sequence: requiring a full-length domain alignment). Restoring glocal alignment to HMMER3 should improve search performance for profiles that are expected to match over their entire length, such as Pfam protein domain models. However, the fast E-value statistics for Forward and Viterbi scores (including MSV filter scores) are only valid for local alignment, and the numeric underflow analysis of the sparse rescaling technique in the Forward/Backward implementation assumes local alignment. Both problems will need to be addressed before glocal alignment is implemented usefully in HMMER3.

Here I have only described single-core performance. I have not discussed parallelization across multiple cores. HMMER3 search programs include rudimentary implementations of POSIX threads and MPI parallelization (message-passing in a cluster of computing nodes). These implementations currently scale poorly, to only modest numbers of processor cores (2–4 for multithreading, for example). Improved parallelization is a priority for future development.

HMMER3's handling of biased composition sequences is problematic. I chose to introduce an *ad hoc* “bias filter” into the acceleration pipeline, to deal with a small number of profiles that let too many sequences through the MSV filter and bog down in the slow Forward/Backward stages of the pipeline. The bias filter occasionally filters true positive hits. A disturbing failure mode can occur when a target sequence consists of a homologous subsequence surrounded by a large amount of nonhomologous biased composition sequence; in this case, the bias filter may aggressively remove the entire sequence. Although other database search programs have analogous issues with over-aggressive composition masking, one future focus for HMMER3 development will be on improving its formal probability model of *non*homologous sequence.

This paper describes an initial baseline for HMMER3 speed performance on a single processor core. The prospects for substantial future improvements are good. There are many obvious opportunities for incremental optimizations. Bjarne Knudsen (CLCbio, Aarhus, Denmark) has already contributed an important optimization of the MSV filter that increases overall HMMER3 speed by about two-fold. The Knudsen optimization will appear in the next HMMER code release, and we will likely describe it in a future manuscript. Another optimization opportunity is to preprocess the target database sequence file into an efficient binary format, as BLAST does with its BLAST databases. HMMER3 still reads standard flatfile sequence databases, such as FASTA and UniProt text formats. Another optimization opportunity is to convert the filters in the pipeline from their current mode of filtering entire target sequences (which was easy to implement) to instead store and retrieve more information about the location of alignment probability mass, so subsequent steps (including Forward/Backward) can be done as banded dynamic programming calculations within high-probability envelopes, as opposed to reprocessing the entire query/target comparison at each pipeline step. Because of these and other straightforward optimization opportunities, I expect HMMER3 speed will surpass NCBI BLAST speed in the relatively near future.

This speed makes it feasible to apply profile HMM technology to standard sequence database searches with single sequence queries, including iterative database searches. A position-independent scoring system for single sequences is just a special case of a profiled position-specific scoring system. A “profile” HMM can be built from a single sequence, using position-independent probabilities obtained from standard scoring matrices such as BLOSUM62, plus a couple of parameters for gap-open and gap-extend probabilities. The HMMER3 software package includes a program *phmmer* for protein database searches akin to *blastp*, and a program *jackhmmer* for iterative protein database searches akin to *psiblast*. These programs, their parameterization, and the effect of extending profile HMM technology, the Forward algorithm, and probabilistic inference methods to routine sequence database searches will be described elsewhere.

## Materials and Methods

### Software implementation and availability

HMMER3 is implemented in POSIX ANSI/ISO C99. Vector implementations are provided for Intel-compatible (SSE) and PowerPC (Altivec/VMX) processors. A “dummy” non-vectorized implementation is provided for other processors, sufficient to enable compilation but about 100× slower than normal HMMER3 vectorized performance. HMMER3 is portable to any POSIX-compliant operating system, including Linux and Mac OS/X, and also to Windows if an optional POSIX compatibility package has been installed (such as Cygwin). It builds from source using a standard GNU configure script and UNIX make. It includes a suite of automated tests written in Perl, C, and Bourne shell. User documentation is provided as PDF and man pages. Source code is freely available under a GNU GPLv3 license. Precompiled binary distributions are available for several platforms including Intel/Linux, Intel/MacOSX, and Intel/Windows. A web interface to HMMER3-based database searches is available, including batch searches and RESTful web services, hosted on computational resources supported by the Howard Hughes Medical Institute. All these resources are at http://hmmer.janelia.org.

### Software and database versions used

Software versions used: SAM 3.5 (Jul 2005) [37], NCBI BLAST+ 2.2.24+ (Aug 2010) [3], FASTA 36.3.3 (Feb 2011) [40], WU-BLAST 2.0MP-WashU (May 2006), HMMER 2.3.2 (Oct 2003), and HMMER 3.0 (Mar 2010).

Example sequence alignments and profile HMMs were sampled from Seed alignments and profiles in Pfam 24 [11]. Example target sequences were sampled from UniProt version 2011_03 [43]. One experiment that characterized roundoff error used older versions, Pfam 22 and UniProt 7.0.

### Speed benchmarking

All program timings were measured in total (wall clock) time on a single execution thread (single core) of a dedicated and unloaded cluster node, where a node has eight 2.66 GHz Intel Gainestown X5550 cores and 24 GB RAM. The same search was run twice sequentially and timed in the second run, to allow filesystem caching of target databases.

For speed benchmarks of programs that take a single sequence as a query (instead of an alignment), the median length sequence was extracted from the query alignment.

### Construction of the sensitivity/specificity benchmark

Sensitivity/specificity benchmarks were created with the create-profmark program, included in the HMMER3 source code. This program allows construction of the wide range of different and randomized benchmarks used during HMMER development. My concern is that because benchmarking is repeated at every step of code development, it is nearly impossible for a developer to avoid overtraining on any in-house benchmark. Synthesizing a variety of partially randomized benchmarks helps mitigate this effect somewhat, compared to relying on a single static benchmark.

The profmark benchmarks use a set of trusted multiple alignments (such as Pfam Seed alignments) as a source of both *query multiple alignments* and distantly related *true test sequences* trusted to be homologous. The individual sequences in each input alignment are clustered by pairwise percent identity, and different clusters are selected to be queries versus test sequences such that no true test domain has more than 25% identity to any sequence in the query alignment, and no true test domain has more than 50% identity to another true test domain. To create realistic-length true test sequences, and to challenge the ability of a program to detect homologous local alignments in a larger target sequence, true test sequences are synthesized by embedding one or two test domains in a larger nonhomologous sequence.

Using a sequence database like Pfam instead of a 3D structure database like SCOP or CATH as a source of trusted true homology relationships has the advantage that a more challenging variety of sequences is tested. Structure databases are biased toward well-ordered globular domains. A weakness of a sequence-based benchmark is that “true homologs” are inferred by current computational sequence comparison methods, rather than being defined by an independent criterion like 3D structure comparison. In particular, my profmark benchmarks are constructed from Pfam alignments as a trusted definition of true homologs, and Pfam is itself constructed with HMMER. There may be some danger that this circularity creates a bias against other search programs. Specifically the danger is that if there are remote homologs that are undetected by profile HMM methods, but that could be detected by another method, any such sequences have been selected against in Pfam. Intuitively, I think this danger is negligible. If a search program was sufficiently powerful that it could detect homologs excluded from Pfam, it ought to be even better at detecting the closer homologs that were included and artificially separated (by profmark's clustering procedure) into challengingly dissimilar query alignment and true test domains. Empirically too, any danger has seemed negligible, because profmark benchmarks tend to be broadly concordant with other published benchmarks [28], [44]. Nonetheless, I have more confidence in using profmark benchmarks for internal comparisons (HMMER vs. HMMER, for different option settings) than for comparisons to other search programs.

False (decoy) sequences (including the nonhomologous flanking sequence around embedded test domains in true test sequences) are created synthetically. If we are trying to find methods that detect previously undetectable homologies, no source of real biological sequences will ever be reliably known to be nonhomologous to the benchmark, and we certainly do not want to penalize a powerful method that identifies new true relationships that are currently annotated as nonhomologous “false positives” [29]. One disadvantage of synthetic nonhomologous sequence is that it is difficult to create realistic sequences with the same challenging properties of real biological sequences, such as biased composition and repetitive sequence.

In detail, the profmark creation procedure is the following, starting from a source of multiple alignments (usually Pfam seeds) and a source of typical single target sequences (usually UniProt/SwissProt):

- Convert all degenerate residue characters to X. (Although HMMER reads all standard degeneracy codes for protein and nucleic acid sequences, some search programs do not.)
- Remove sequence fragments. By default, any sequence of length less than 70% of the mean unaligned sequence length in the alignment is defined as a fragment.
- Cluster the sequences by single-linkage clustering at a default threshold of 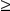
25% percent identity (defined as the number of identical residues divided by the shorter sequence length, in the given pairwise alignment). Between any two clusters, there is no pair of sequences closer than 25% identity. If there is only one cluster, exclude this alignment from the benchmark and skip to the next alignment. Define the largest cluster as the *query*. Save it to a file, in its original multiple alignment.
- Cluster the remaining sequences by single-linkage clustering at a default threshold of 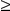
50% identity. If there are less than two clusters, exclude this alignment from the benchmark and skip to the next alignment. From each cluster, select one sequence at random. These are the *true test domains*.
- Create synthetic true test sequences by embedding one or two true test domains in a larger nonhomologous sequence. The total length of the test sequence is sampled from the length distribution of the input sequence database, conditional on being at least as long as the true test domain(s). True test domains are inserted at randomly sampled locations in the sequence. The remaining two or three nonhomologous sequence segments are synthesized as described below. Thus true test sequences are composed either of three segments (one homologous, two not) or five segments (two homologous, three not).
- The program implements a choice of several different methods for generating nonhomologous sequence segments. The default is “monoshuffling”: to select a sequence segment at random from the input sequence database and shuffle its residues, preserving 0th-order residue composition and bias. Figure 6 also shows the use of i.i.d. (independent identically distributed) synthetic sequence with each residue simply sampled from the average residue frequency distribution of proteins. Other options include reversed sequences and shuffling while preserving di-residue composition. Though more realistic, and useful when looking carefully and manually for failure modes, di-residue shuffling and reversed sequences are problematic as a source of nonhomologous segments in automated benchmarking. Exact di-residue shuffling preserves significant sequence identity to the original sequence over surprising segment lengths, and reversed sequences are surprisingly significantly more likely to show a significant match to the original sequence (because of a counterintuitive statistical effect of the frequency of approximate palindromes in any sequence).
- Decoys (negative sequences) are created by randomly selecting a true test sequence (solely to obtain its three or five segment lengths – not its sequence) then concatenating nonhomologous segments of the same lengths. The length distribution of negative sequences, and the length distribution of potentially biased nonhomologous subsequences embedded in them, is therefore matched to the distributions for the true test sequences.

For the experiments in Figure 6, the create-profmark procedure was applied to 11,912 Pfam 24 seed alignments [11] and the UniProt/SwissProt sequence database (version 2011_03, 516,081 sequences) [43] either with options –iid –mono (Figure 6A) or default (Figure 6B). The benchmark in panel A is composed of 2,141 query alignments, 24,040 true test sequences containing single homologous domains, and 200,000 decoys. The benchmark in panel B is composed of 2,141 query alignments, 11,547 true test sequences containing two homologous domains, and 200,000 decoys.
